# Expression Profiles of Cuproptosis-Related Genes Determine Distinct Subtypes of Pancreatic Ductal Adenocarcinoma

**DOI:** 10.3390/curroncol30020126

**Published:** 2023-01-29

**Authors:** Yusheng Chen, Xuan Zou, Mingjian Ma, Yu Liu, Ruijie Wang, Zhengjie Dai, Yesiboli Tashiheng, Yu Yan, Xianjun Yu, Xu Wang, Chen Liu, Xuan Lin, He Cheng

**Affiliations:** 1 Department of Pancreatic Surgery, Fudan University Shanghai Cancer Center, Shanghai 200032, China; 2 Department of Oncology, Shanghai Medical College, Fudan University, Shanghai 200032, China; 3 Shanghai Pancreatic Cancer Institute, Shanghai 200032, China; 4 Pancreatic Cancer Institute, Fudan University, Shanghai 200032, China

**Keywords:** pancreatic ductal adenocarcinoma, cuproptosis, prognostic evaluation, cell death, overall survival

## Abstract

Background: Pancreatic ductal adenocarcinoma (PDAC) is the most prevalent subtype of pancreatic cancer and one of the most malignant tumors worldwide. Due to the heterogeneity of its genomics and proteomics, the prognosis of PDAC remains disappointing despite advances in surgery and medicines. Recently, a novel form of programmed cell death, cuproptosis, was proposed, although its role in PDAC has not been investigated. This study aimed to quantify the expression of cuproptosis-related genes and characterize the novel subtypes of PDAC. Methods: To evaluate the pattern of cuproptosis in PDAC, the gene expression data and clinical information of 372 samples were collected from the Cancer Genome Atlas (TCGA) and Gene Expression Omnibus (GEO) databases. A consensus cluster analysis was performed using the transcriptional levels, genetic alterations, and individual prognostic values of seven pre-selected cuproptosis-related genes (*DLAT*, *LIPT1*, *FDX1*, *DLD*, *PDHB*, *PDHA1*, and *LIAS*) to identify the novel subtypes associated with cuproptosis in PDAC. A univariate Cox regression analysis was used to determine the significant prognostic indicators and cuproptosis scores among the differentially expressed genes (DEGs) between the dividing subclusters, followed by a principal component analysis. The prognostic values, immune profiles, treatment sensitivities, and cuproptosis scores were evaluated between the different subgroups. Results: Seven cuproptosis-related genes showed aberrant expression levels and genetic alterations in the PDAC tumor microenvironment. Among them, *LIPT1*, *LIAS*, *DLAT*, *PDHA1*, and *DLD* were significantly correlated with overall survival. Based on the expression profiles of the seven cuproptosis-related genes, three cuproptosis clusters (Clusters A, B, and C) were identified, which were represented by different clinicopathologic features, gene expression levels, and biological processes. A total of 686 DEGs were identified among the three cuproptosis clusters, of which 35 prognosis-related DEGs were selected to further classify the PDAC samples into two subgroups with different survival rates, clinicopathologic features, immune infiltration levels, and drug sensitivities. Higher cuproptosis scores were associated with a significantly poorer prognosis. Conclusion: The cuproptosis subtypes, scores, and relevant genes represent valuable information for assessing the heterogeneity, treatment, and prognosis of PDAC.

## 1. Introduction

Pancreatic ductal adenocarcinoma (PDAC) is a highly aggressive malignancy with a disappointing prognosis [1]. PDAC accounted for 3.2% of all new cancer cases in the U.S. in 2022. The overall five-year survival rate for the years 2012–2018 was 11.5%, according to data published by the National Cancer Institute [2]. Advances in PDAC treatment strategies over the past few years have resulted in slightly improved outcomes, although the prognosis remains poor [3]. Genomics and proteomics have become significant tools for depicting the heterogeneity of PDAC [4]. Public genomic data concerning PDAC tumor biology may serve as useful resources for improving the diagnosis, prognosis, and treatment response, deciphering drug resistance, and developing new drugs.

Copper ions serve as a co-factor for essential enzymes and play a critical role in biological processes [5]. However, the intracellular copper concentrations need to be maintained at low levels, and excessive copper accumulation triggers cytotoxicity [6]. Tumor cells may take in more copper, as a higher copper concentration was found in serum and tumor tissues [7,8,9]. A retrospective study by Marcin et al. demonstrated a relationship between elevated serum Cu levels and pancreatic cancer incidence, which indicated Cu to be a marker for diagnosis and possible development [10]. In the spontaneous pancreatic cancer model, mice treated with CuSO4 in their drinking water developed significantly larger pancreatic tumors [11]. Copper, as a co-factor for pro-angiogenic factors and the relevant oncogenic signaling pathways, also contributes to tumor development [12,13]. Thus, the modulation of the intracellular copper levels (Cu ionophores, Cu chelators, and stable Cu complexes) has emerged as an attractive novel therapeutic target for cancer therapeutics [14,15].

The mechanism by which copper overload leads to cytotoxicity was unclear until a recent study showed that copper-induced cell death is mediated by mitochondrial ferredoxin 1-mediated protein lipoylation [16]. This new form of regulated cell death (RCD) induced by copper was defined as “cuproptosis”. Briefly, the direct binding of copper to the components of the lipoylated tricarboxylic acid (TCA) cycle results in lipoylated protein aggregation, which leads to subsequent proteotoxic stress and, ultimately, cell death [17]. It has been reported that cuproptosis-related genes (*FDX1, LIPT1, LIAS, DLD, DLAT, PDHA1*, and *PDHB*) may predict the prognosis and response to immunotherapy [18,19,20,21].

In this work, we investigated the clinical relevance of cuproptosis genes and established the cuproptosis-related subtypes in PDAC. A consensus clustering analysis identified three clusters (Clusters A, B, and C) based on the cuproptosis gene expression patterns in PDAC, of which Cluster B displayed a favorable prognosis. A total of 35 prognosis-related DEGs among the clusters were selected to divide the PDAC samples into two more discriminative subgroups (geneClusters A and B) with significant survival differences. The cuproptosis scores in the PDAC patients were then calculated. Significant differences in the survival rates, clinicopathologic features, immune infiltration levels, and drug sensitivity profiles were observed between the high and low cuproptosis score groups. Higher cuproptosis scores were associated with a significantly poorer prognosis. Overall, our study for the first time indicated the clinical significance of the newly discovered form of RCD, cuproptosis, in PDAC, which may provide new clues for the treatment and prognostic evaluation of PDAC.

## 2. Method

### 2.1. Data Sources and Processing Methods 

Three independent datasets (TCGA-PAAD (*n* = 178), GSE21501 (*n* = 132), and GSE57495 (*n* = 63)) that contain gene expression data and clinical information concerning PDAC patients were analyzed. In the TCGA-PAAD dataset, the level 3 RNA sequencing (RNA-seq) data were downloaded using the GDC Data Transfer Tool. The human reference genome (GRCh38 version) was used to annotate the official gene symbols. Data concerning the survival status, follow-up time, and clinicopathologic features of the PDAC patients were also obtained. Two GEO studies of the expression profiling array using PDAC tumor tissues were further analyzed. For each study, the clinical information of the PDAC patients and the gene expression levels detected by probes were extracted from the corresponding series matrix file separately. The platform file of each array was downloaded to convert the probe IDs to gene symbols. To generate a comprehensive gene expression matrix, the batch effects of the three datasets were removed using the “limma” and “sva” R packages. After removing the batch effects, a matrix recording the expression levels of the 15,557 protein-encoded genes of the combined 372 samples was used for further analysis. 

### 2.2. Online Tools Exploring Gene Expression Patterns, Genetic Alterations, and Gene Methylation Levels

The expression levels of the cuproptosis-related genes in the different cell types of PDAC were explored via TISCH (http://tisch.comp-genomics.org, accessed on 5 January 2023), an online source enabling the visualization of single-cell transcriptome profiles from published tumor databases [22]. For PDAC, two datasets (CRA001160 and GSE111672) were analyzed. Multi-omics data concerning PDAC, including the single nucleotide variant (SNV) frequencies, heterozygous and homozygous copy number variation (CNV) statistics, and gene methylation and expression levels, were analyzed using GSCALit (http://bioinfo.life.hust.edu.cn/web/GSCALite/) (accessed on 5 January 2023), a web server providing comprehensive and visualized analyses of cancer multi-omics data from the TCGA and Genotype-Tissue Expression (GTEx) [23]. 

### 2.3. Principal Component Analysis (PCA)

A PCA is an approach for dealing with high-dimensional data, such as transcriptome data, by reducing the data dimensions and extracting representative principal components (PCs) [24]. In this study, a PCA was conducted using the “FactoMineR” and “factoextra” R packages to detect the existence of batch effects, evaluate the sample discrimination based on the characteristic gene expression profiles, and derive an index score from several gene signatures to classify the samples into distinct subgroups. For the sample classification, the cuproptosis scores were calculated as the sum of the first two critical PCs in the PCA (PC1 + PC2), and the optimal cutoff value of the PC scores that could significantly distinguish high- and low-risk PDAC patients was determined using Cutoff Finder [25]. 

### 2.4. Cancer Subtypes Identified via the Consensus Clustering Method

In this study, the 372 PDAC samples were divided into several clusters based on the gene expression matrix data using the consensus clustering method, which has gained popularity in cancer genomics research [26,27,28]. In the present study, the “ConsensusClusterPlus” R package was applied for the k-means clustering of the dataset matrix, as previously described [29]. When running “ConsensusClusterPlus”, we selected 80% item resampling (pItem), 80% gene resampling (pFeature), a maximum evaluated k of 9 so that cluster counts of 2, 3, 4, 5, 6, 7, 8, and 9 were evaluated (maxK), and 50 resamplings (reps). The consensus cumulative distribution function (CDF) plot, delta area plot, tracking plot, and consensus matrices for each k-value were used as outputs to determine the optimal k-value with the best isomorphism. According to the developers’ instructions [29], the optimal k-value should meet the following criteria: (1) the CDF reaches an approximate maximum, meaning that the consensus and cluster confidence is at a maximum at this k; (2) there is no appreciable increase in the consensus at this k compared with k-1; (3) the k yields the cleanest consensus matrix with the most obvious cluster partition; and (4) there is no cluster with a particularly small sample size.

### 2.5. Identification of Differentially Expressed Genes (DEGs)

Based on the expression profiles of the cuproptosis-related genes, the 372 PDAC samples were divided into several clusters, and then differential gene expression analyses were conducted between each cluster for further functional exploration. The curated gene expression levels from the combined matrix were compared between each cluster using the “limma” R package. DEGs were identified when logFC > 0.5 and FDR < 0.05. The “clusterprofiler” R package was further applied to depict the linkages between the DEGs and biological functions, such as the Gene Ontology (GO) items and Kyoto Encyclopedia of Genes and Genomes (KEGG) pathways.

### 2.6. Gene Set Variation Analysis (GSVA)

A GSVA was performed to compare the potential functions of the different cuproptosis-related clusters using transcriptome data and the “GSVA” R package [30]. The classical Hallmark and KEGG pathway gene sets obtained from the MSigDB database (https://www.gsea-msigdb.org/gsea/msigdb, accessed on 5 January 2023) were analyzed based on the gene expression profiles. The normalized enrichment score of each sample was calculated for each gene set. The enrichment scores were compared using the “limma” R package to identify the most differentially expressed pathways between two clusters. Moreover, the relative proportions of the tumor-infiltrating immune cells were further estimated by assessing the enrichment scores of each immune cell-type signature using the “ssGSEA” function of the “GSVA” R package. 

### 2.7. Prediction of Chemotherapeutic Response

The “pRRophetic” R package was used to predict the drug response from the gene expression data [28]. Briefly, statistical models generated from the gene expression data and drug sensitivity data of the cell lines were applied to the gene expression data of the tumor specimens to yield drug sensitivity predictions. The half maximal inhibitory concentration (IC50) values were predicted to measure the drug potency in PDAC. Drugs with significantly lower IC50 values were considered to be sensitive for PDAC treatment.

### 2.8. Statistical Analyses

The Mann–Whitney *U* test and Kruskal–Wallis *H* test were conducted to compare the gene expression levels, immune cell infiltration degrees, PC scores, and drug IC50 values across the different subgroups. A Spearman correlation analysis was performed to evaluate the correlation of the gene expression levels as well as the correlation between the PC scores and immune cell infiltration degrees. The Kaplan–Meier method was used to compare the overall survival of the PDAC patients between the different subgroups. A univariate Cox regression analysis was conducted to evaluate whether the gene expression level was a prognostic factor for PDAC. All the statistical analyses and graph construction were performed using R 4.1.1 (https://www.r-project.org, accessed on 5 January 2023) and SPSS 25.0 (SPSS Inc., IL, USA) software. A two-tailed *p*-value < 0.05 was considered to be significant.

## 3. Results

### 3.1. Gene Expression and Genetic Alterations of Cuproptosis-Related Genes in PDAC

**Figure 1:**
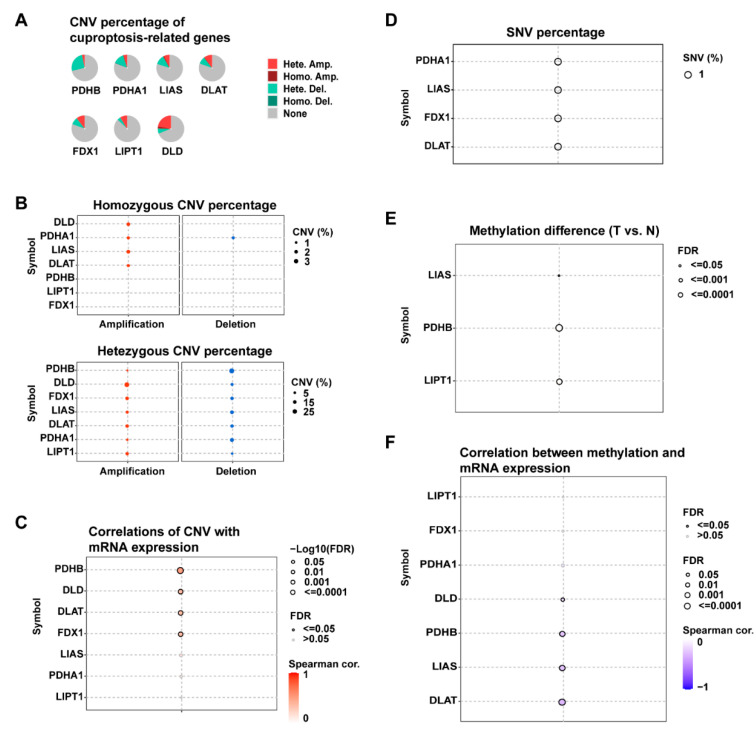
Genetic alterations of cuproptosis-related genes in PDAC. (**A**,**B**) Pie plots and bubble plots showing the percentages of CNV types for each gene in the 178 PDAC tumor samples of the TCGA-PAAD dataset; CNV: copy number variation; Hete. Amp: heterozygous amplification; Homo. Amp: homozygous amplification; Hete. Del: heterozygous deletion; Homo. Del: homozygous deletion. (**C**) Correlation between the gene expression levels and CNV values; FDR: false discovery rate. (**D**) Bubble plot summarizing the frequencies of the deleterious mutations of cuproptosis-related genes reported in the TCGA-PAAD dataset; SNV: single nucleotide variant. (**E**) Bubble plot showing the differences in the gene methylation levels between the PDAC tumor samples and normal samples; T: tumor, N: normal. (**F**) Correlation between the gene methylation and mRNA expression levels.

To investigate the cuproptosis-related gene expression in PDAC, we selected seven cuproptosis regulators (*DLAT, LIPT1, FDX1, DLD, PDHB, PDHA1,* and *LIAS*) and analyzed their average gene expression levels in the PDAC tissues of different cell types. According to two single-cell RNA datasets (CRA001160 and GSE111672), in addition to malignant cells, these genes also showed distinct expression in the immune cells and stromal cell types in the tumor microenvironment, such as B cells and fibroblasts (Supplementary Figure S1). The presence of genetic alterations, including the SNVs and CNVs of these cuproptosis-related genes, was further analyzed using multi-omics data from the TCGA-PAAD dataset (Figure 1). In PDAC, SNVs of these genes were rare, with mutation rates below 1%, although CNVs were more prevalent (Figure 1A). High frequencies of heterozygous deletion CNV were observed in *PDHB*, *PDHA1*, and *LIAS*, whereas heterozygous amplification CNVs were more common in *DLAT*, *FDX1*, *LIPT1*, and *DLD* (Figure 1B). In addition, a correlation analysis revealed that the CNVs were significantly correlated with the gene expression of *PDHB*, *DLD*, *DLAT*, and *FDX1* in PDAC (FDR < 0.05) (Figure 1C). The DNA methylation of the cuproptosis-related genes was also analyzed. As shown in Figure 1E,F, the methylation levels of *LIAS*, *PDHB*, and *LIPT1* in the PDAC tumor tissues were significantly different from those in the normal tissues (FDR < 0.05). Significant impacts of the DNA methylation levels on the gene expression could be observed in *DLD*, *PDHB*, *LIAS*, and *DLAT* (FDR < 0.05).

### 3.2. Cuproptosis-Related Genes Were Significant Prognostic Factors for PDAC

**Figure 2:**
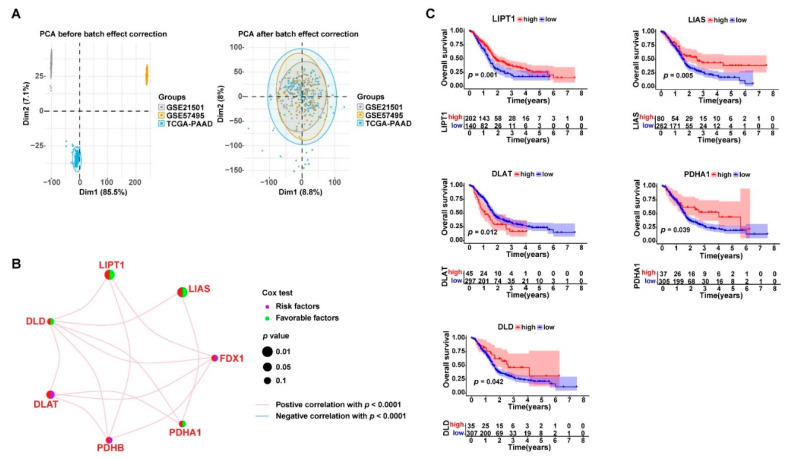
Cuproptosis-related gene expression was closely associated with the prognosis of PDAC. (**A**) Three transcriptome sequencing datasets, TCGA-PAAD (*n* = 177), GSE21501 (*n* = 132), and GSE57495 (*n* = 63), were combined for further analysis (*n* = 372) after removing the batch effects using the “limma” and “sva” R packages; the left PCA plot shows the large dispersion difference among the samples from the three datasets before removing the batch effects; the right PCA plot shows that the samples were successfully clustered together after the batch effect correction; PCA: principal component analysis. (**B**) Network plot displaying the expression correlations between the genes; the association between the gene expression and PDAC prognosis is annotated by dots in different colors according to the univariate Cox regression analysis. (**C**) Kaplan–Meier survival curves showing the association between the gene expression levels and PDAC prognosis.

Three gene expression datasets (TCGA-PAAD, GSE21501, and GSE57495) were combined for analysis. The PCA analysis showed that the batch effects were reduced from the gene expression data of the three datasets (Figure 2A). Therefore, the corrected gene expression profiles of a total of 372 PDAC samples were used for further analysis. As shown in Figure 2B, significant positive correlation in the gene expression was widely observed among the seven cuproptosis-related genes (*p* < 0.001). The univariate Cox regression analysis revealed that the *DLAT* expression was a significant risk factor for PDAC prognosis, whereas *LIAS* and *LIPT1* were significantly favorable factors (*p* < 0.05). The Kaplan–Meier survival analysis further demonstrated that there was a strong association between the expression levels of the cuproptosis-related genes and the overall survival of the PDAC patients (Figure 2C). The PDAC patients with higher expression levels of *PDHA1*, *LIPT1*, *LIAS*, and *DLD*, or lower expression levels of *DLAT*, in their tumor tissues had a better prognosis than their counterparts (*p* < 0.05).

### 3.3. Consensus Clustering Based on Cuproptosis-Related Genes Expression Identified Three Clusters in PDAC

**Figure 3:**
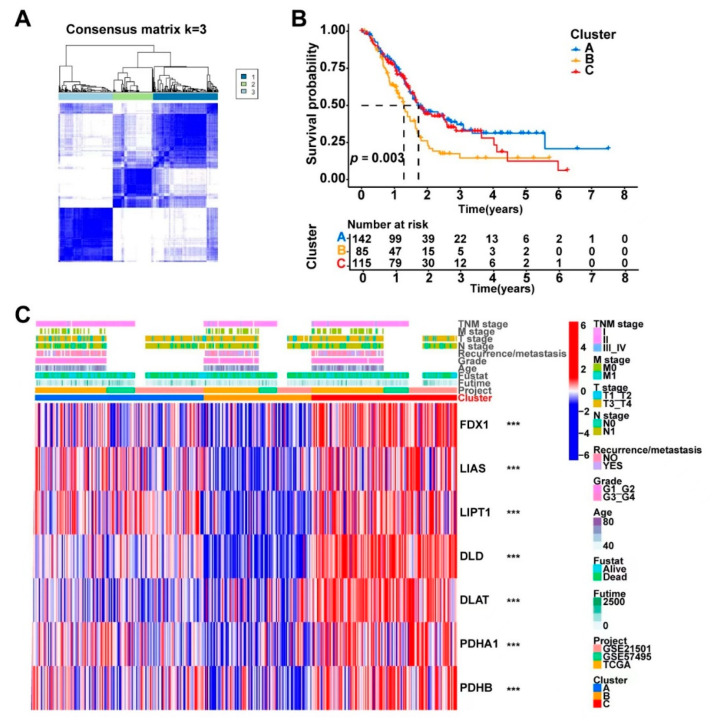
The combined 372 PDAC samples were divided into three main clusters using the consensus clustering method based on the expression levels of the cuproptosis-related genes. (**A**) Consensus matrix of the optimal cluster number (k = 3). (**B**) Kaplan–Meier survival analysis comparing the patients’ prognosis between the three clusters (Clusters A, B, and C). (**C**) Heatmap displaying the distribution of the clinicopathologic features and gene expression levels of each sample in the three clusters; the expression levels of the cuproptosis-related genes were compared between the three clusters; *** *p* < 0.001. A PCA analysis based on transcriptome data showed that the three identified clusters had distinct differential gene expression patterns (Figure 4A). To better understand the functional differences between the three clusters, we performed a differential gene expression analyses using the “limma” R package. A number of DEGs (logFC > 0.5 and FDR < 0.05) were identified between Clusters A, B, and C (Supplementary Figure S5A). A combination of these DEGs was then generated and found to contain a total of 686 protein-coding genes. The potential biological functions of these 686 DEGs generated by comparing the different cuproptosis-related clusters were further explored via a GO (Supplementary Figure S5B) and KEGG (Figure 4B,C) pathway enrichment analysis. The 686 DEGs were significantly enriched in several classical cancer-related pathways, such as the “PI3K-Akt signaling pathway”, “PPAR signaling pathway”, “EMC-receptor”, and “Proteoglycans in cancer”, which indicated that cuproptosis was associated with cancer development in PDAC.

We identified three distinct clusters (Clusters A, B, and C) from the consensus clustering analysis of the 372 PDAC samples based on the expression profiles of the cuproptosis-related genes (Figure 3A). As shown in Figure S2, k = 3 reached the maximum consensus according to the CDF plot and the delta area plot, and the consensus matrix plots further showed that k = 3 indicated the cleanest distribution of samples, so the three-cluster pattern was chosen to divide the samples. The PDAC patients from the three clusters had significantly different survival statuses according to the Kaplan–Meier survival analysis (Figure 3B). The distribution of the clinicopathologic features and gene expression levels of each sample in the three identified clusters is shown in Figure 3C. The seven cuproptosis-related genes also had significantly varied expression levels across the three clusters. The pathway activity was compared between the different clusters via GSVA, and the results indicated that the three clusters had obviously different characteristics in terms of the biological processes (Supplementary Figure S3). Since the cuproptosis-related genes were widely expressed in the tumor-infiltrating immune cells in PDAC, the relative infiltrating degrees of 23 immune cell types were calculated via ssGSEA and compared among the three clusters. The PDAC samples from the different cuproptosis-related clusters had significantly different infiltration levels of several key immune cell types, including activated CD4+ T cells, activated CD8+ T cells, CD56dimNK cells, eosinophils, neutrophils, and T helper 2 (TH2) cells, indicating a potential role of cuproptosis in PDAC cancer immunology (Supplementary Figure S4).

In addition, the underlying role of non-coding RNAs in cuproptosis in PDAC should not be ignored. We further conducted differential gene expression analyses for both protein-coding genes and non-coding genes based on the transcriptome data from the TCGA-PAAD dataset (Supplementary Table S2). Among the 60,245 genes included in the analysis, a total of 3201 genes were differentially expressed between Clusters A, B, and C (FDR < 0.05 and FC > 2 or FC < −2). The functional enrichment analysis further revealed the potential correlation between these DEGs and some metabolism-related pathways and cancer-related pathways.

### 3.4. The DEGs in the Three Clusters Separated the PDAC Samples into Two Subgroups with Significantly Different Survival and Tumor Immune Status 

**Figure 5:**
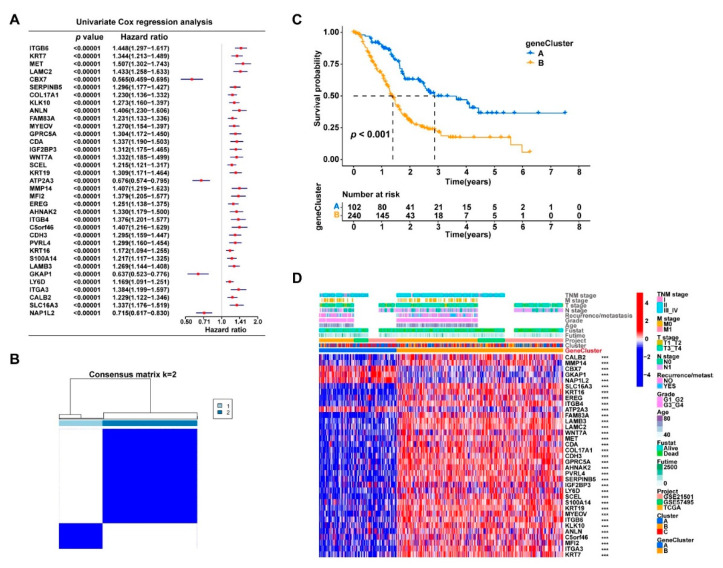
The prognosis-related DEGs from the three cuproptosis-related clusters could further divide the PDAC samples into two subgroups with significant survival difference. (**A**) The univariate Cox regression analysis identified 35 DEGs that were the most significant prognostic factors (*p* < 0.00001) for PDAC patients; the *p*-values and hazard ratios with 95% confidence intervals are presented in the forest plot.(**B**) The 372 PDAC samples were clustered into two subtypes (geneClusters A and B) based on the 35 gene expression levels using the consensus clustering algorithm; the optimal k value was 2. (**C**) Kaplan–Meier survival curves comparing the patients’ survival between geneClusters A and B. (**D**) Heatmap displaying the distribution of the clinicopathologic features and gene expression levels of each sample in geneClusters A and B; gene expression levels of the cuproptosis-related genes were compared between the three clusters; *** *p* < 0.001.

According to the results of the univariate Cox regression analysis, nearly half of the identified DEGs (331/686) were potential prognostic factors for PDAC (*p* < 0.05, Supplementary Table S1), which further demonstrated the prognostic value of cuproptosis in PDAC. A total of 35 DEGs were the most significant prognostic indicators, with *p* < 0.001 (Figure 5A). We attempted to divide the 372 PDAC samples into more discriminative subgroups via consensus clustering based on the expression profiles of these 35 genes. Two subgroups (geneClusters A and B) with the cleanest consensus matrix plot were identified (Figure 5B), which displayed significant survival differences (*p* < 0.001, Figure 5C). The output figures of the consensus clustering are provided in Supplementary Figure S6. Figure 5D displays the distribution of the clinicopathologic features of the samples and the significant gene expression differences between the two clusters. The expression levels of these 35 genes were all significantly different between geneClusters A and B (*p* < 0.001).

**Figure 6:**
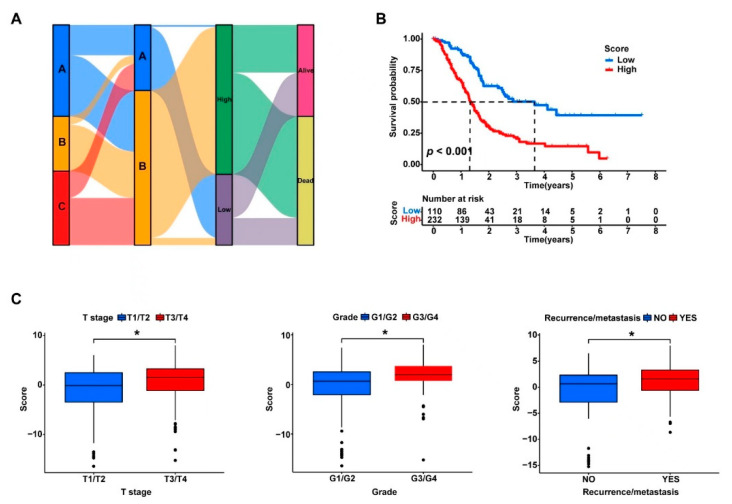
The PC scores based on the expression levels of the 35 prognosis-related DEGs were strongly associated with the survival of the PDAC patients and the tumor immune status. (**A**) The expression profiles of the 35 DEGs were clustered via PCA, and the PC scores of each sample were calculated as the sum of PC1 and PC2 with the maximum variances; The Sankey plot shows the corresponding network of the three cuproptosis-related clusters (Clusters A, B, and C), the two subgroups based on the prognosis-related DEGs (geneClusters A and B), the two groups with high and low PC scores, and the sample groups with different survival outcomes (dead or alive); PCA: principal component analysis; PC: principal component. (**B**) A Kaplan–Meier survival analysis was conducted to compare the overall survival between the patients with high and low PC scores. (**C**) The PC scores were compared between the patients with different T stages, tumor grades, and recurrence or metastasis statuses. * *p* < 0.05.

Briefly, the PDAC samples were grouped into Clusters A, B, and C based on the expression profiles of the cuproptosis-related genes, and then the 35 prognosis-related DEGs from the three clusters were identified and the PDAC samples were further divided into two more discriminative subgroups (geneClusters A and B). To facilitate the quantification of the subgrouping method, a PCA analysis was further performed using the expression data of these 35 genes for the dimensionality reduction, and the cuproptosis scores (PC1 + PC2) were calculated for the quantitative analysis. The optimal survival data-based cutoff value of the cuproptosis scores was −0.5734 according to CutoffFinder. The 372 PDAC samples were finally divided into two groups with high or low cuproptosis scores using the determined cutoff value. The corresponding network of the three cuproptosis-related clusters (Clusters A, B, and C), the two subgroups based on the prognosis-related DEGs (geneClusters A and B), the two groups with high and low cuproptosis scores, and the sample groups with different survival outcomes (dead or alive) are presented in Figure 6A. More clearly, the PDAC patients with higher cuproptosis scores had significantly worse survival than those with lower scores (*p* < 0.001, Figure 6B). Consistently, the PDAC patients with larger tumor sizes (T3/T4 vs. T1/T2), higher tumor grades (G3/G4 vs. G1/G2), and tumor recurrence or metastasis also had significantly higher cuproptosis scores (*p* < 0.05, Figure 6C).

Furthermore, the link between the cuproptosis score and tumor immune status remained strong. The calculated cuproptosis scores were significantly correlated with the tumor infiltration levels of most immune cell types, and several immune checkpoints may have differential expression levels between the high- and low-score subgroups (Supplementary Figure S7). 

### 3.5. Drug Sensitivity Profiles of the Two Cuproptosis-Related PDAC Subgroups 

**Figure 7:**
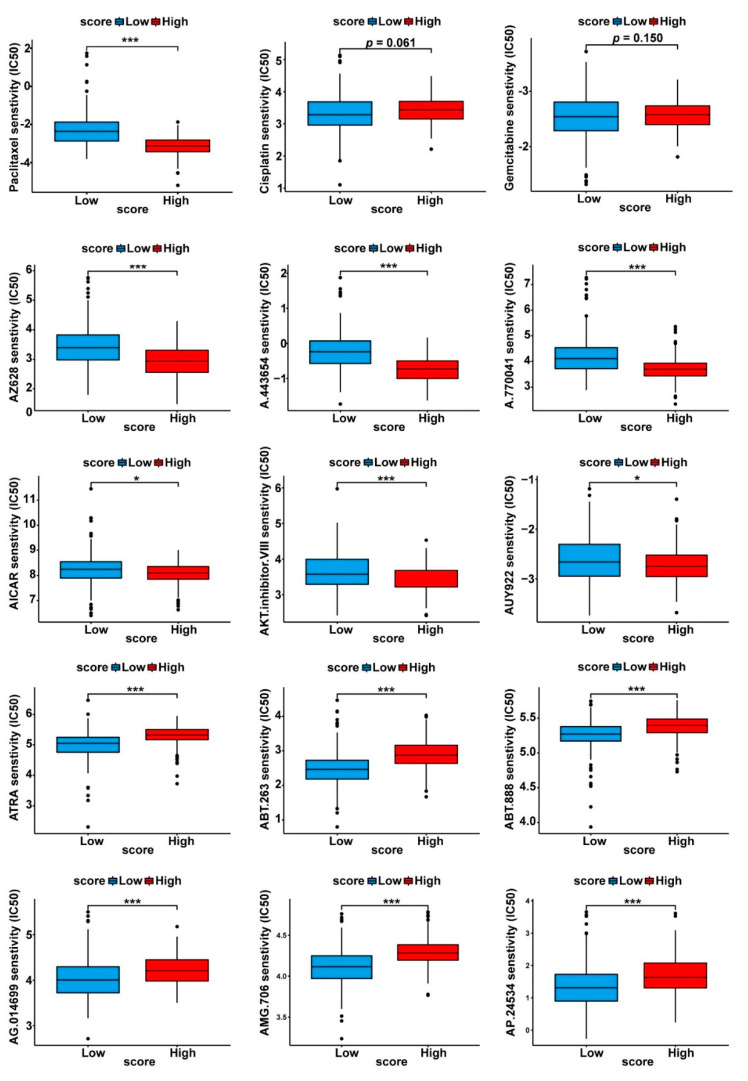
Prediction of the chemotherapeutic response between the subgroups with high and low PC scores. The phenotype of the chemotherapeutic response for each sample was predicted from the gene expression data using the “pRRophetic” R package; the drug sensitivity (IC50) values were calculated, and the drugs with the most significant response differences between the two subgroups are shown in the box plots; drugs with significantly lower IC50 values were considered to be sensitive for PDAC treatment. * *p* < 0.05, *** *p* < 0.001.

The “pRRophetic” method enabled the prediction of the drug response from the gene expression data [31]. By comparing the drug sensitivity data between the high- and low- cuproptosis score subgroups, we sought to identify potentially effective drugs for PDAC treatment. As shown in Figure 7, among the most commonly used therapeutic modalities for PDAC treatment (such as paclitaxel, gemcitabine, and cisplatin), paclitaxel turned out to have significantly different responses between the high- and low-score subgroups. Figure 7 also lists some of the most sensitive drugs for the two subgroups, including AZ628, AICAR, and ATRA.

## 4. Discussion

The morbidity and mortality of PDAC have increased in recent years, and the treatment of PDAC remains challenging. Traditional chemotherapies, targeted therapies, and immune checkpoint blockade therapies have limited efficacy and are unsatisfactory. Therefore, the treatment, prevention, and prognostic evaluation of PDAC must take into account its genetic background and tumor microenvironment. A newly discovered type of regulated cell death, cuproptosis, was recently proposed [16].

Copper is essential for supporting cell development and replication by participating in a wide range of metabolic processes, such as aerobic respiration, iron transport, and superoxide dismutation [32]. However, elevated concentrations of free ionic copper in cytoplasm may be toxic. Recent studies have identified specific functions of copper metabolism in oncogenesis controlled by biological processes such as mitogenic respiration, immune system regulation, and autophagy [15,33]. Notably, according to recent findings, excessive copper may sensitize cells toward the death pathway. Such copper-induced cell death differs from previously known forms of programmed and nonprogrammed cell death, and it is defined as “cuproptosis”. Copper can induce cell death by targeting the lipoylated components of the tricarboxylic acid cycle, resulting in proteotoxic stress and, ultimately, cell death [16]. The associations between cuproptosis and cancer development, disease prognosis, and drug sensitivity are still to be determined. An in-depth understanding of the roles of copper homeostasis and cuproptosis in tumors may facilitate copper-dependent diagnostic and therapeutic strategies for improving patient outcomes, although the connection between cuproptosis and PDAC remains largely unknown.

Cell death plays a key role in cancer cell elimination. Cuproptosis is a new pattern of programmed cell death that is mediated by mitochondrial ferredoxin 1-mediated protein lipoylation. Signatures based on cuproptosis have showed substantial predictive values for different tumors, such as breast cancer, prostate cancers, as well as cell renal carcinomas [34,35,36,37]. Such studies found that the cuproptosis-related signature could predict the prognosis and indicate the status of the immune microenvironment in tumors, which could help guide personalized immunotherapy treatment for patients. In pancreatic cancer, Jiang et al. [37] identified a cuproptosis-related lncRNA signature associated with the PDAC prognosis. In our study, we systematically analyzed the landscape of the gene alterations of cuproptosis in pancreatic cancer. The subgroups based on cuproptosis showed different survival rates, clinicopathologic features, immune infiltrating levels, and drug sensitivities. The cuproptosis-related risk score showed good performance in terms of prognostic prediction in PDAC. Thus, our findings strongly suggested that the cuproptosis-related subtypes and relevant genes may facilitate the prediction of the prognosis and the design of personalized treatments for pancreatic cancer.

This study comprehensively analyzed the expression patterns and clinical significance of the cuproptosis-related genes in PDAC. We found that in PDAC, several cuproptosis-related genes showed specific genomic variation characteristics, with CNV events being relatively more frequent and closely associated with the mRNA expression levels. Moreover, most of these cuproptosis-related genes were significantly associated with overall survival in the PDAC patients. Based on the expression levels of the cuproptosis-related genes, we identified three distinct clusters via a consensus clustering analysis with significantly different expression of the cuproptosis-related genes and immune cell infiltration levels of PDAC. A total of 686 DEGs were identified among the three clusters, and these genes were particularly enriched in some canonical cancer-related pathways. In addition, the three cuproptosis-related clusters also exhibited obvious distinctions in the patient outcomes, laying the foundation for identifying the cuproptosis-relevant subtypes with different prognoses in PDAC patients. 

A consensus clustering analysis based on the prognostic DEGs further classified the PDAC patients into two more distinct clusters with more pronounced survival differences. To facilitate clinical application, we further performed a PCA analysis and calculated the PC scores for the survival prediction of the PDAC patients. According to the optimal cutoff value of the PC scores, the PDAC patients were sub-divided into the high- and low-score groups. The two subgroups showed significant differences in the patient outcomes and clinicopathologic features. Furthermore, the immunophenotypes, including the tumor immune cell infiltration levels and immune checkpoint expression levels, were also significantly varied between the two distinct subgroups. In conclusion, the cuproptosis-related genes contributed to the identification of valid subgroup models, which deepened the link between cuproptosis and PDAC development.

Finally, a drug sensitivity analysis was further performed to explore the therapeutic implications for PDAC. We observed that the final two subgroups responded differently to certain cancer drugs, including AZ628 (RAF inhibitor), A.443654 (AKT inhibitor), A.770041 (Src family Lck inhibitor), AICAR (AMPK activator), AUY922 (HSP-90 inhibitor), ABT.888 and AG.014699 (PARP inhibitors), AMG.706 (VEGFR inhibitor), and AP.24534 (a pan-BCR-ABL inhibitor). Further investigations are needed to explore the relationship between the cuproptosis-related subtype systems and the treatment responses in PDAC.

In conclusion, this study systematically analyzed the expression patterns and genomic alterations of the cuproptosis-related genes in PDAC. A subtype scoring system developed based on the cuproptosis-related genes is helpful in distinguishing PDAC patients with different prognoses and clinicopathologic features. The cuproptosis-related subtypes may have different immunological characteristics and drug susceptibility spectrums, suggesting the complex influences of cuproptosis on PDAC tumor biology. 

## Figures and Tables

**Figure 4:**
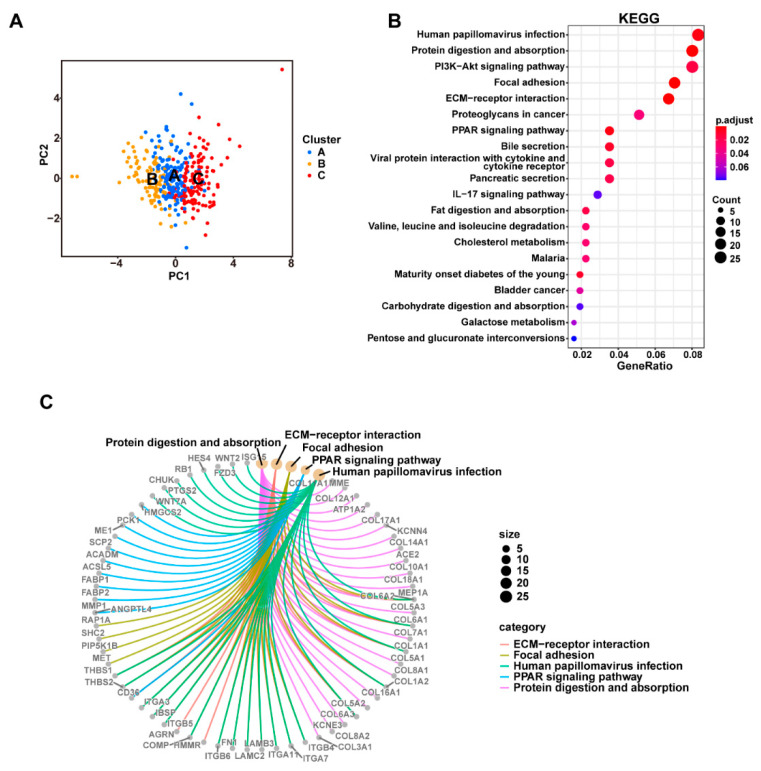
The three cuproptosis-related clusters had distinct differences in their gene expression patterns and biological functions. (**A**) Samples of the three clusters could be well-separated based on the PCA of the gene expression data; PCA: principal component analysis. (**B**) KEGG pathway enrichment analyses of all 686 DEGs between the three clusters; bubble plot shows the KEGG pathways with the most significant differences; the counts of genes enriched in each pathway are represented by the dot sizes; DEG: differentially expressed genes; KEGG: Kyoto Encyclopedia of Genes and Genomes. (**C**) Network showing the DEGs enriched in the five most significant pathways.

## Data Availability

All the transcriptome data generated or analyzed during the present study was downloaded from the TCGA (https://portal.gdc.cancer.gov, accessed on 5 January 2023) and GEO (https://www.ncbinlm.nih.gov/geo, accessed on 5 January 2023) databases that could be available freely with open access.

## Supplementary Materials

The following supporting information can be downloaded at: https://www.mdpi.com/article/10.3390/curroncol30020126/s1, Figure S1. Average expression of the cuproptosis-related genes in the different cell types of PDAC. The heatmaps display the gene expression levels in various malignant, immune, and stromal cell types of PDAC according to two single-cell RNA datasets (CRA001160 and GSE111672). Tprolif: proliferating T cells; CD8T: CD8+ T cells; CD8Tex: exhausted CD8+ T cell; B: B cells; Plasma: plasma cells; DC: dendritic cells; Mono/Marco: monocytes or macrophages; Mast: mast cells; Endothelial: endothelial cells; Malignant: malignant cells; Acinar: acinar cells; Ductal: ductal cells; Endocrine: endocrine cells; Stellate: stellate cells. Figure S2. Output figures of the consensus clustering analysis that divided the samples into three clusters. (A) Delta area plot. (B) Consensus cumulative distribution function (CDF) plot. (C) Tracking plot. (D) Consensus matrices for each k-value. Figure S3. Heatmaps of the GSVA analyses exploring the functional differences between the three cuproptosis-related clusters. Gene sets of the HALLMARK and KEGG pathways were downloaded from the Msigdb database, and the pathways with the most significant enrichment differences are shown. Figure S4. Box plots comparing the infiltration levels of various immune cells in the tumor tissues between the three cuproptosis-related clusters. The Y-axis is the infiltration degree of the immune cells in the tumor tissues predicted via ssGSEA; * *p* < 0.05, ** *p* < 0.01, *** *p* < 0.001, ns: not significant. Figure S5. DEGs between the three cuproptosis-related clusters were identified. (A) Volcano plots displaying the differentially expressed genes (DEGs) between Cluster A and B, Cluster A and C, and Cluster B and C; FC: fold change. (B) GO function annotation of all 686 DEGs; bubble plots show the samples of the GO terms with the most significant differences; the counts of the genes enriched in each process or pathway are represented by the dot sizes; GO: Gene ontology, BP: biological process, CC: cellular component, MF: molecular function. Figure S6. Output figures of the consensus clustering analysis that divided the samples into two gene clusters. (A) Delta area plot. (B) Consensus cumulative distribution function (CDF) plot. (C) Tracking plot. (D) Consensus matrices for each k-value. Figure S7. The PC scores based on the expression levels of the 35 prognosis-related DEGs were strongly associated with the tumor immune status of the PDAC patients. (A) Correlation heatmap displaying the correlation between the PC scores and infiltration levels of various immune cells in the tumor tissues; dots in red represent positive correlation, while dots in blue represent negative correlation; * *p* < 0.05. (B) Box plots comparing the expression levels of several typical immune checkpoints between the high- and low-PC score subgroups.
