# Supplementary figures and images for: Expression Profiles of Cuproptosis-Related Genes Determine Distinct Subtypes of Pancreatic Ductal Adenocarcinoma
Source: Curr Oncol. 2023 Jan 29;30(2):1648–62. doi: 10.3390/curroncol30020126 (PMC9955227; doi:10.3390/curroncol30020126)

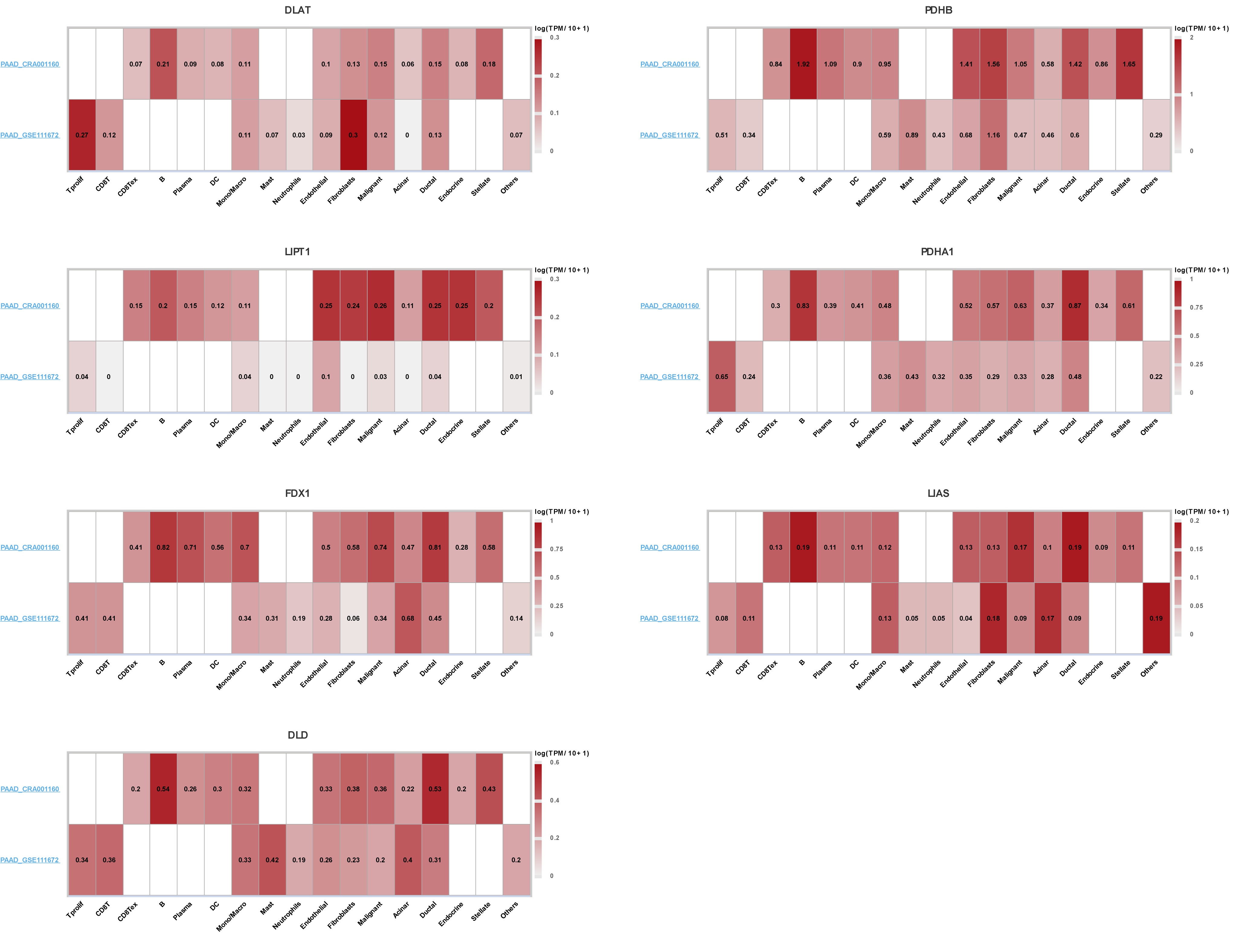

Supplement: Supplementary file 1 [file curroncol-30-00126-s001.zip › Figure S1.tif]

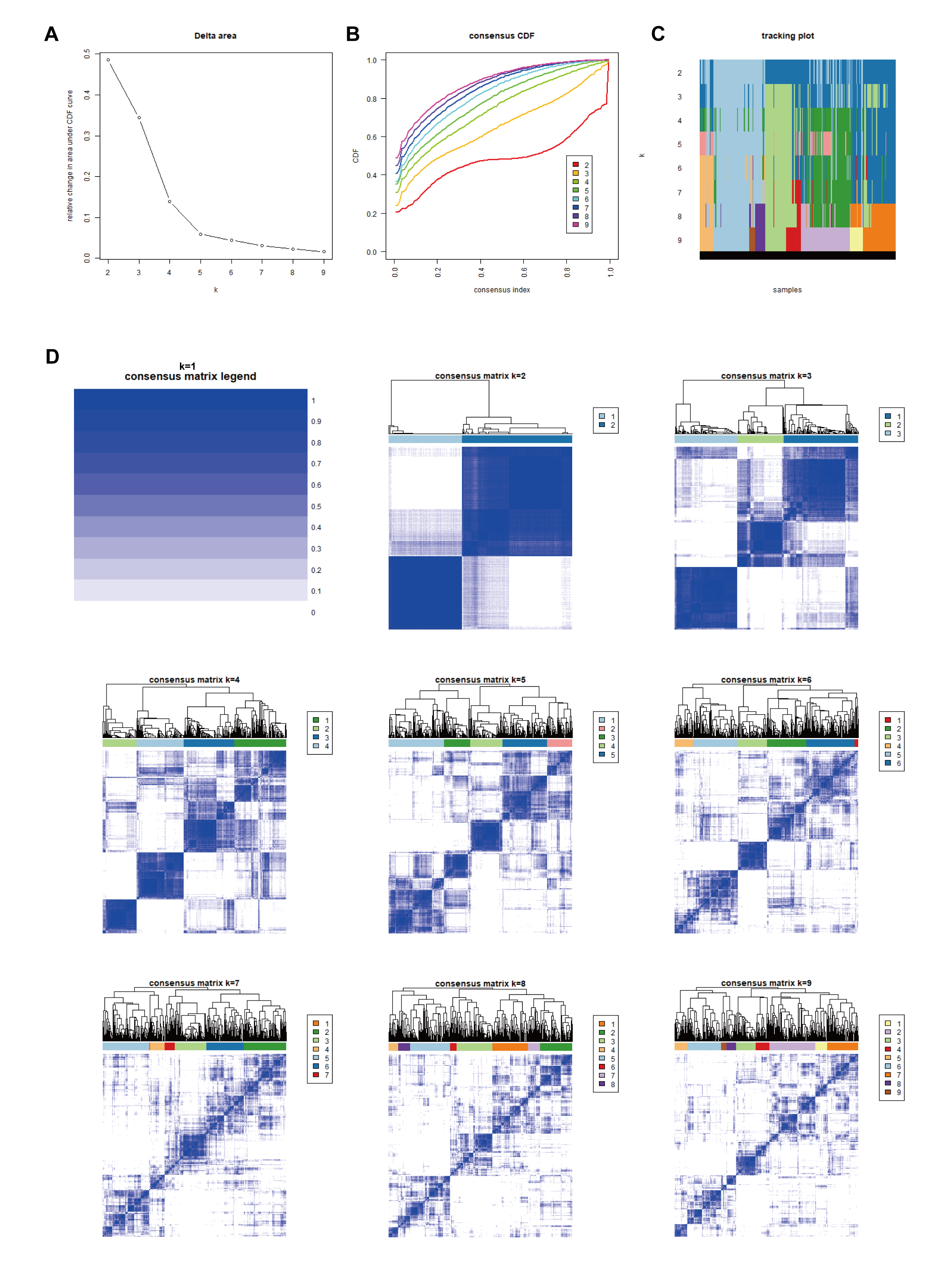

Supplement: Supplementary file 1 [file curroncol-30-00126-s001.zip › Figure S2.tif]

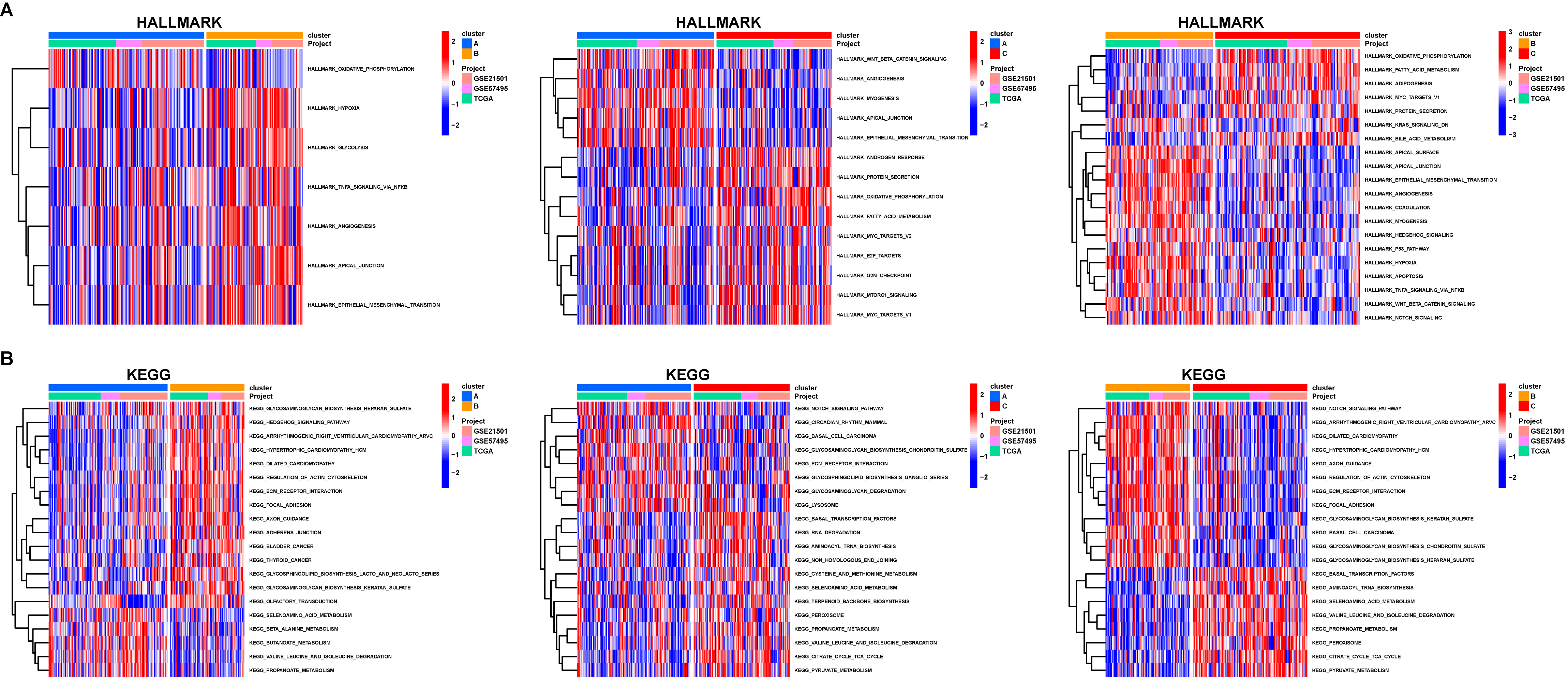

Supplement: Supplementary file 1 [file curroncol-30-00126-s001.zip › Figure S3.tif]

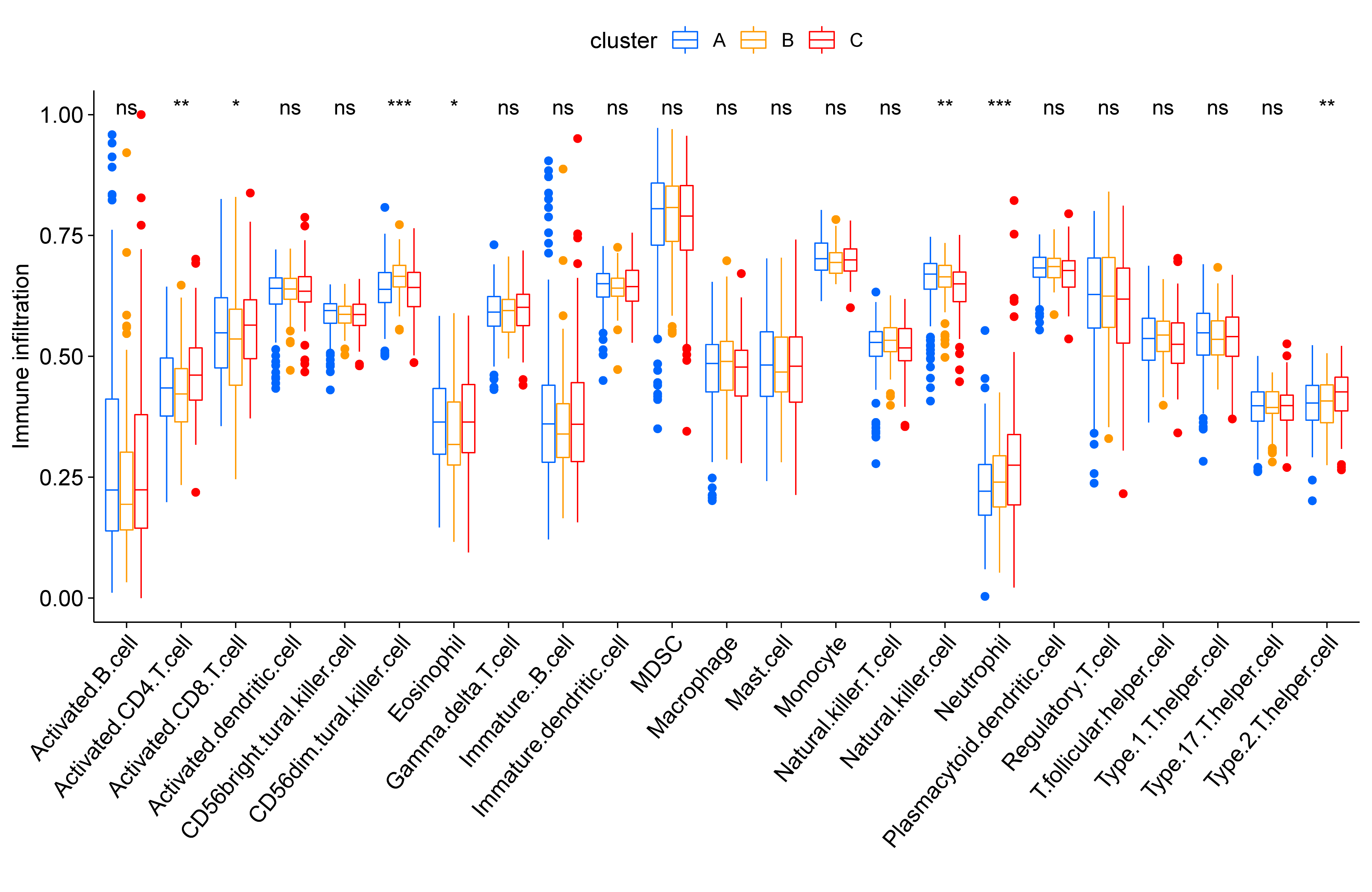

Supplement: Supplementary file 1 [file curroncol-30-00126-s001.zip › Figure S4.tif]

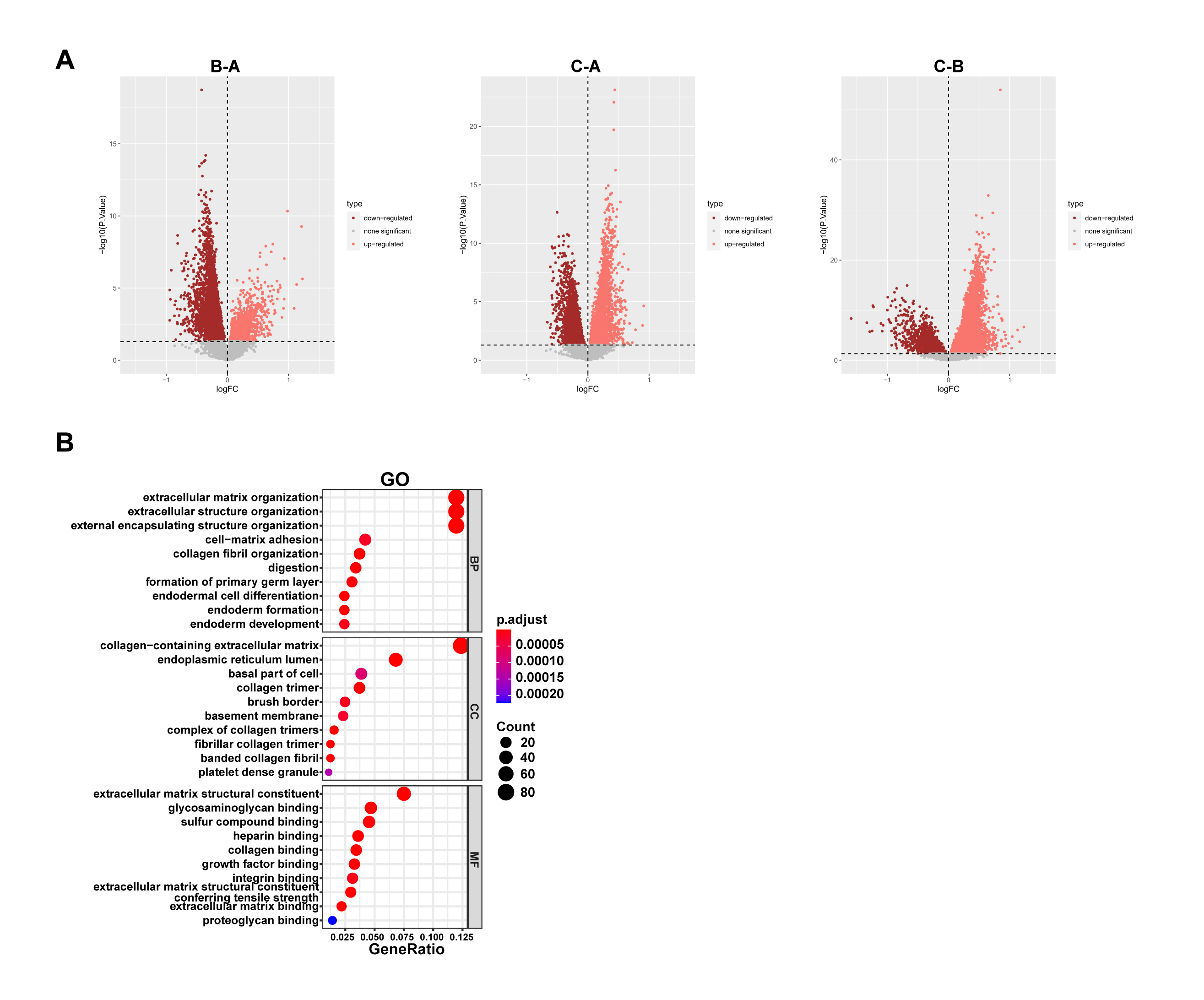

Supplement: Supplementary file 1 [file curroncol-30-00126-s001.zip › Figure S5.tif]

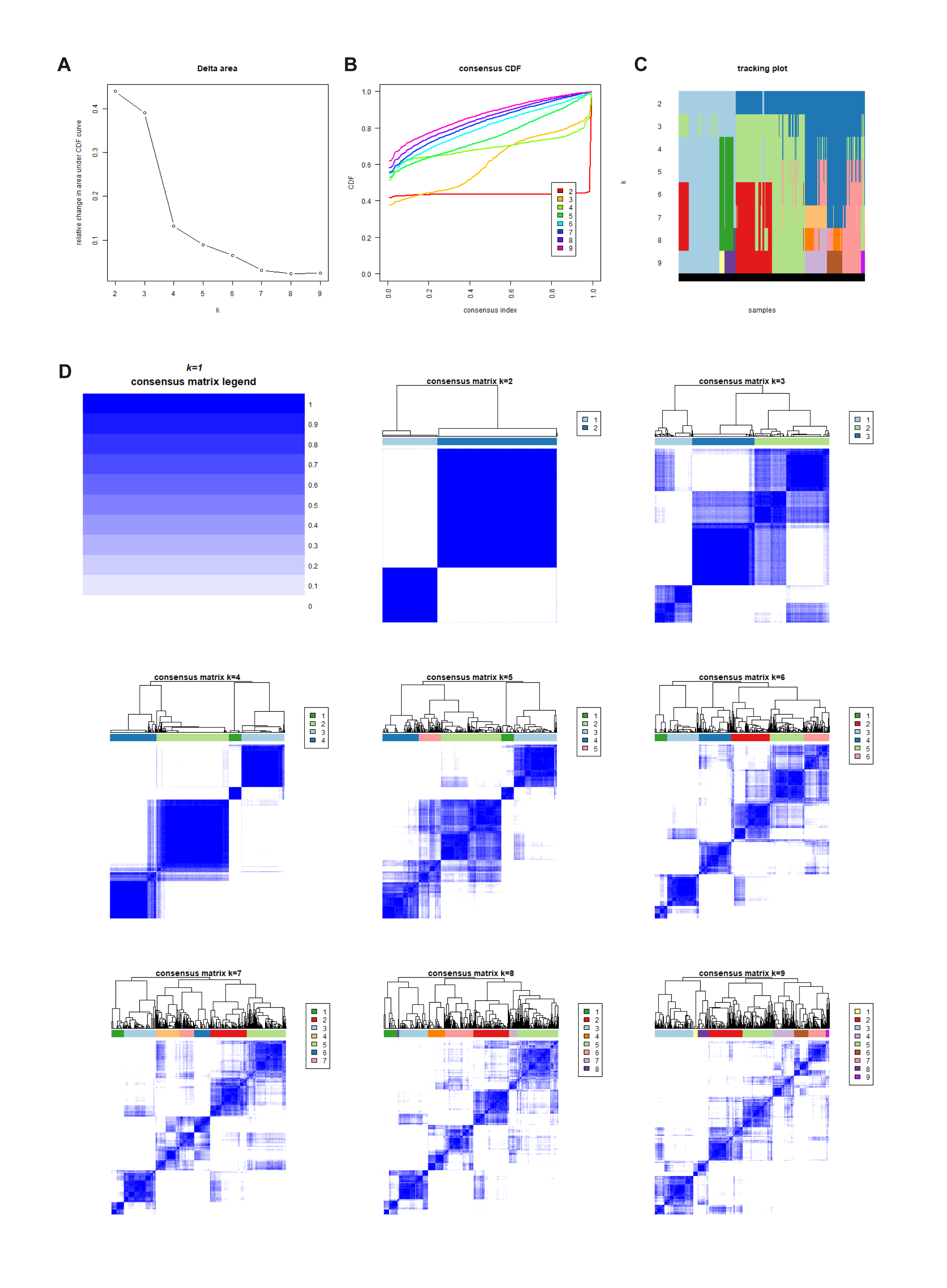

Supplement: Supplementary file 1 [file curroncol-30-00126-s001.zip › Figure S6.tif]

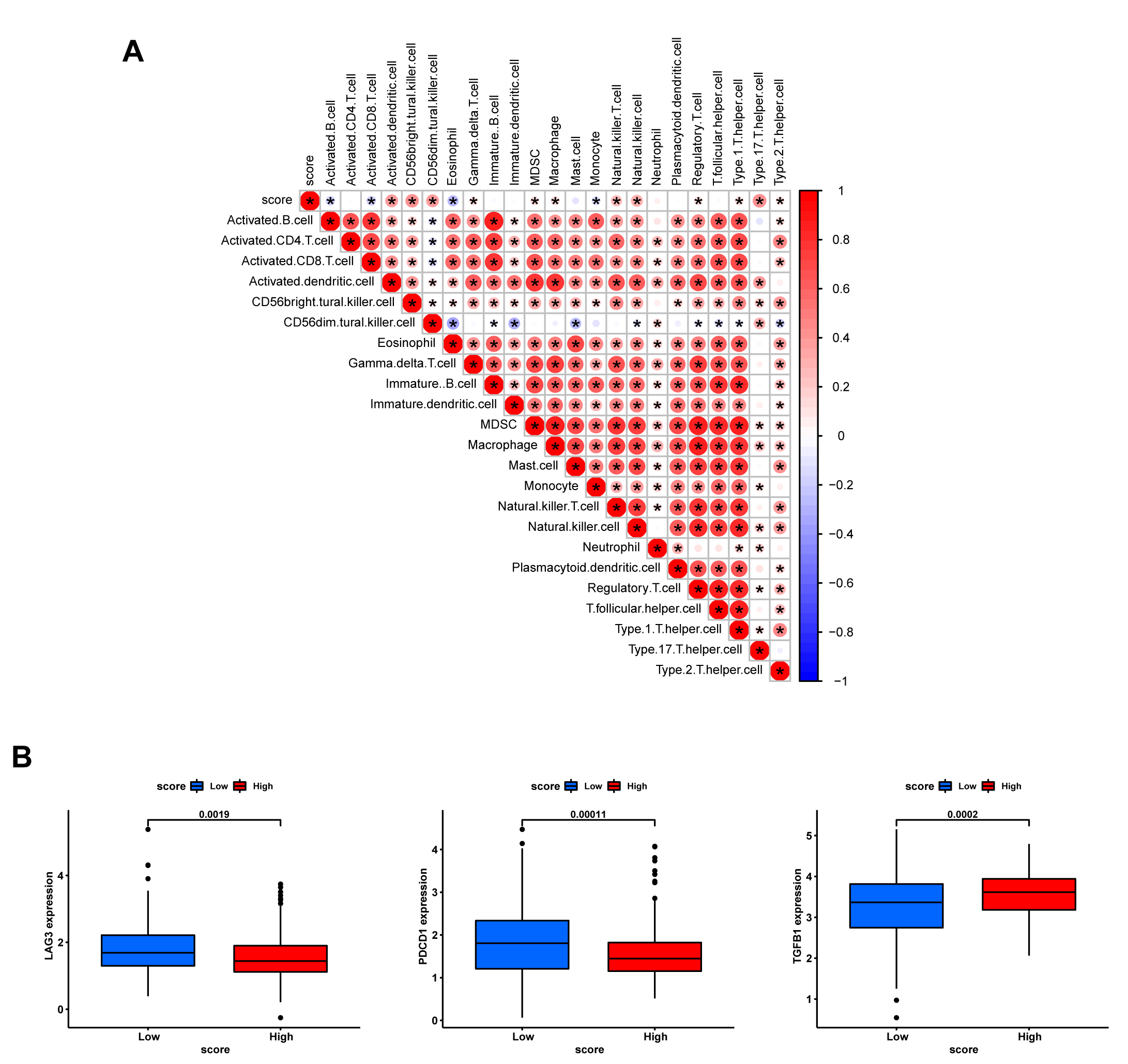

Supplement: Supplementary file 1 [file curroncol-30-00126-s001.zip › Figure S7.tif]
